# Effectiveness of *Panax ginseng* on Acute Myocardial Ischemia Reperfusion Injury Was Abolished by Flutamide via Endogenous Testosterone-Mediated Akt Pathway

**DOI:** 10.1155/2013/817826

**Published:** 2013-10-24

**Authors:** Luo Pei, Hou Shaozhen, Dong Gengting, Chen Tingbo, Liu Liang, Zhou Hua

**Affiliations:** ^1^State Key Laboratory for Quality Research of Chinese Medicine, Macau University of Science and Technology, Avenida Wai Long, Taipa, Macau, China; ^2^School of Chinese Pharmaceutical Science, Guangzhou University of Chinese Medicine, University Town, Guangzhou 510006, China

## Abstract

Mechanisms for *Panax ginseng*'s cardioprotective effect against ischemia reperfusion injury involve the estrogen-mediated pathway, but little is known about the role of androgen. A standardized *Panax ginseng* extract (RSE) was orally given with or without flutamide in a left anterior descending coronary artery ligation rat model. Infarct size, CK and LDH activities were measured. Time-related changes of NO, PI3K/Akt/eNOS signaling, and testosterone concentration were also investigated. RSE (80 mg/kg) significantly inhibited myocardial infarction and CK and LDH activities, while coadministration of flutamide abolished this effect of RSE. NO was increased by RSE and reached a peak after 15 min of ischemia; however, flutamide cotreatment suppressed this elevation. Western blot analysis showed that RSE significantly reversed the decreases of expression and activation of PI3K, Akt, and eNOS evoked by ischemia, whereas flutamide attenuated the effects of these protective mechanisms induced by RSE. RSE completely reversed the dropping of endogenous testosterone level induced by I/R injury. Flutamide plus RSE treatment not only abolished RSE's effect but also produced a dramatic change on endogenous testosterone level after pretreatment and ischemia. Our results for the first time indicate that blocking androgen receptor abolishes the ability of *Panax ginseng* to protect the heart from myocardial I/R injury.

## 1. Introduction

Ischemic heart disease (IHD) is one of the most common cardiovascular diseases, and it is often caused by occlusion and reperfusion of the coronary artery. Present studies have demonstrated that the prevention of damage induced by myocardial ischemia reperfusion (I/R) injury is the key to successful therapy for IHD [1]. In the past few years, gender disparity in myocardial response to acute I/R injury has been reported, and the difference is considered to be hormone(s) mediated [2]. Recent studies have demonstrated that estradiol and its metabolites are the major biologically active players in this action, and they protect the heart and blood vessels in multiple ways [3]. In contrast to estrogen, androgen's role in cardiac disease remains unclear [4, 5]. The relationship between androgens and cardiovascular disease is controversial due to numerous conflicting clinical and epidemiological studies [6]. For example, androgen deprivation in prostate cancer therapy leads to higher mortality due to cardiovascular failure [7]. Administration of testosterone (a primary and most well-known androgen) can reduce myocardial ischemia in patients with coronary artery disease [8]. And low endogenous testosterone levels have been correlated with several risk factors for increased myocardial infarction [9]. It is therefore believed that androgen plays beneficial effects in cardiovascular diseases. However, testosterone had also been suggested to exhibit fewer antioxidant effects in cardiac muscle and to worsen cardiac function in mice suffering from myocardial infarction [10]. Zaugg et al. also carried out exogenous androgen supplementation in adult myocytes and demonstrated increase of apoptosis in rat ventricular cells [11]. This evidence nevertheless supports that androgen may have detrimental effects in cardiovascular diseases and have given rise to the popular belief that, after a myocardial infarction, men are more likely than women to die on the way to the hospital [12]. Whether androgen supplement is beneficial or harmful is thus hard to determine. Consequently, more work is needed to elucidate the role of endogenous androgen in acute I/R injury which results in the most significant morbidity in cardiovascular diseases.

Ginseng is the root of *Panax ginseng* C.A. Meyer. It is now cultivated in China, Korea, Japan, and Russia. The name *Panax* means “all cure,” which describes the traditional conception that ginseng has power to heal all the disorders of the body [13]. Ginseng contains more than 30 types of ginsenosides, and it is these components to which most of the pharmacological actions of ginseng can be attributed. Recently, ginseng-derived herbal medicines and supplements have been studied for their ability to protect against myocardial I/R injury in various animal species and human objects. Studies by us and others suggest that these pharmaceutical effects are probably mediated by sex hormones and their related receptors, such as the glucocorticoid receptor (GR) or estrogen receptor (ER) [14]. While female hormones have been studied, male hormones have not, even though there is evidence for the role of androgen in the cardioprotective effect of ginseng. In this report, we investigated the role of endogenous androgen on the cardioprotective effect of RSE, a standardized ginseng extract, in an open-chest left anterior descending (LAD) coronary artery ligation rat model. Flutamide, an oral nonsteroidal antiandrogen drug by competing testosterone and its powerful metabolite with androgen receptors, was employed to show the potential role of androgen receptor in the cardioprotective effect of ginseng. 

## 2. Materials and Methods

### 2.1. Animals

Male Sprague-Dawley rats weighing 280–340 g were purchased from the Laboratory Animal Services Center, the Chinese University of Hong Kong, Hong Kong. The animals were acclimated for 7 days under a cycle of 12 hours light and 12 hours dark at room temperature of 22°C ± 1°C. Chow diet and water were provided *ad libitum*. Animal care and treatment procedures were in accordance with the Institutional Guidelines and Animal Ordinance (Department of Health, Hong Kong Special Administrative Region).

### 2.2. Materials

Flutamide (F9397) was purchased from Sigma (St. Louis, MO, USA). 2,3,5-Triphenyltetrazolium chloride (1612634) was from International laboratory USA (SAN BRUNO, CA, USA). Assay kits of lactate dehydrogenase (LDH) and creatine kinase (CK-MB) were obtained from STANBIO (Texas, USA). Nitrate/Nitrite assay kit was purchased from Cayman Chemical (Michigan, USA). The root of *P. ginseng* C.A. Meyer was purchased in a wholesale market in Tonghua county of Jilin Province, China, and authenticated by Professor Ping Ding (an herbalist of Guangzhou University of Chinese Medicine) with quality conforming to the requirements of the Chinese Pharmacopoeia (CP) and Hong Kong Standard of Chinese Materia Medica (HKSCMM). The sample was stored in a desiccated condition in the laboratory until use. Voucher specimens in the form of the whole root were deposited in the State Key Laboratory for Quality Research of Chinese Medicine, Macau University of Science and Technology. 

### 2.3. Preparation of RSE

**Figure 1:**
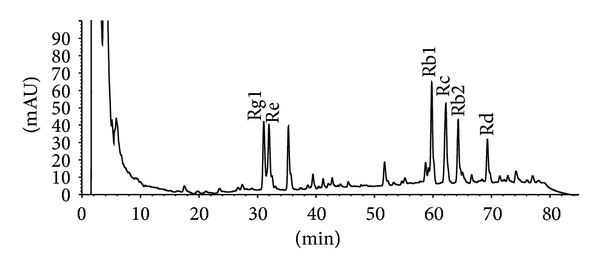
Chromatographic fingerprint of standardized ginseng extract (RSE).

The standardized ginseng extract RSE was prepared by ethanol extraction, a well-established and generally accepted method for preparing ginseng extract. The extraction parameters were optimized in our experiments to obtain a good recovery of major ginsenosides with consistent quality. In brief, the root was refluxed with 5 volumes (versus ginseng weight) of 70% ethanol 3 hr three times. The ethanol extracts were pooled and concentrated at 60°C under vacuum evaporation (0.08 MPa) to half the original volume. The concentrate was finally freeze-dried to powder. The extraction rate was 28%, meaning 1 kg ginseng produced 280 g RSE. To examine the chemical consistency of RSE, the chemical fingerprint (Figure 1) of RSE was established on a Phenomenex ODS column (250 mm × 4.6 mm i.d.; particle size 5 *μ*m; Phenomenex Inc., USA) protected by a Security Guard Cartridge (C18, 4 mm × 3.0 mm i.d.; Phenomenex Inc., USA) by using high performance liquid chromatography (1100, Agilent Technologies, CA, USA) equipped with a G1312A binary pump, G1379A degasser, G1315B diode-array detector, and G1313A autosampler. The mobile phase was acetonitrile (A) and water (B), and the separation was conducted in a gradient manner, in which the ratio of A was 19%, 19%, 29%, 29%, and 40% at 0, 35, 55, 70, and 100 min, respectively. The flow rate was 1.0 mL/min. Detection was conducted at 203 nm. The HPLC fingerprint of RSE is shown in Figure 1. The contents of Rg1, Re, Rb1, Rc, Rb2, and Rd in RSE were 7.63, 6.90, 12.21, 10.65, 7.24, and 4.59 mg/g, respectively.

### 2.4. Experimental Protocol

**Figure 2:**
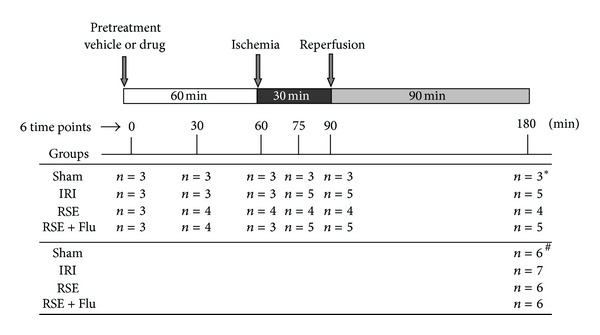
Outline of experimental protocol to evaluate the effects of flutamide on cardioprotection of RSE against myocardial I/R injury in rats. Sham: sham operation group; IRI: I/R injury group with vehicle treatment; RSE, I/R injury with RSE 80 mg/kg treatment; RSE + Flu: I/R injury group with RSE 80 mg/kg + flutamide 10 mg/kg treatment. 6 time points include 0 min, 30 min, and 60 min after pretreatment; 15 min after ischemia; 30 min after ischemia; 90 min after reperfusion. Heart tissue and blood sample were collected, respectively (*n*: number of rats at each time point). ∗: heart and serum samples were collected for Western blot analysis, NO production and testosterone concentration assays. #: heart and serum samples were collected for TTC staining, CK and LDH activities determinations.

Rats were randomly assigned to four experimental groups (shown in Figure 2), that is, Sham group (given vehicle by oral gavage at 60 min before sham surgery without I/R, *n* = 24), I/RI group (given saline by oral gavage at 60 min before I/R, *n* = 31), RSE group (given 80 mg/kg of RSE by oral gavage at 60 min before I/R, *n* = 29), and RSE + Flutamide group (given 80 mg/kg of RSE by oral gavage and 10 mg/kg of flutamide by subcutaneous injection at 60 min before I/R, resp., *n* = 31). Sham group rats were subjected to all the procedures except that the LAD ligation was not tightened. The vehicle or drugs were given 60 min before sham operation or I/R procedure, which was designated as time 0 min. In each group, 3~5 rats were sacrificed at the following time points: 30 min after drug treatment, 1 hr after drug treatment, 15 min after ischemia, 30 min after ischemia, and 90 min after reperfusion. At each time point, arterial blood samples were drawn from aorta immediately before animal sacrifice and then centrifuged at 1,000 g and 4°C for 15 min. All serum samples were stored at −80°C before the measurements of nitrate/nitrite, LDH, and CK. Heart was excised and weighed immediately. After collection of serum samples, 2 mL of 10% potassium chloride was injected via inferior vena cava to stop the heart in diastole, and the heart was excised for infarct size measurement by TTC staining or frozen in liquid nitrogen for Western blot analysis. 

### 2.5. Ischemia Reperfusion Protocol

The ischemia reperfusion injury was produced in rat heart based on Burke's description with modifications [14]. In brief, rats were anesthetized with pentobarbital sodium (70 mg/kg body weight) by an i.p. injection of a mixture of 20% Dorminal (1 mL contains 200 mg pentobarbital sodium, Alfasan) and sterile saline at a ratio of 1 : 3 (v/v). Additional dose (40 mg/kg/h) was administrated continuously via jugular vein by a microinjection pump at an interval of approximately 2 hr or at a rate as required to maintain anesthesia. After ensuring sufficient depth of anesthesia according to the sign of loss of palpebral reflex, the rats were placed on a warm board to control the body temperature at 37°C for surgery. The neck was opened with a ventral midline incision. The trachea was exposed and cannulated with a PE-90 catheter to establish an artificial respiration provided by a SAR-830/P ventilator (IITC, USA) with oxygen at a breath ratio of 1 : 1 and at a frequency of 70~80 breaths/min with tidal volume of 0.8~1.2 mL. The right carotid artery was isolated, and a Millar catheter (Millar Instruments, Inc., Houston, TX, USA) was inserted into the right carotid artery. Using a PowerLab (ADInstruments Pty Ltd., Castle Hill, Australia), mean aortic pressure (MAP) was recorded from the Millar catheter. Electrocardiogram (ECG) in lead II was also recorded through the needle electrodes attached to the limbs. The heart rate and ST-segment elevation were calculated offline. The chest was opened at the left fourth intercostal space. The pericardium was incised, and the left atrium appendage was elevated to expose the LAD coronary artery. A 6-0 silk suture was passed around the LAD coronary artery, and the ends of the suture were threaded through a small vinyl tube to form a snare. The thoracic cavity was covered with saline-soaked gauze to prevent the heart from drying. At 1 hr after drug administration, ischemia was established by tightening the suture from both ends with fixed weight. The animals then underwent 30 min of ischemia, confirmed visually in situ by the appearance of regional epicardial cyanosis and ST-segment elevation. Reperfusion was introduced by releasing the snare gently for a period of 90 min. The sham control animals were subjected to the entire surgical procedures above except the introduction of LAD ligation and release. 

### 2.6. Infarct Size Measurement

Measurement of heart infarct size was performed by TTC staining method as described previously [15]. Briefly, the left ventricle was cut perpendicular to the base-apex axis into six 2-3 mm slices. The slices were incubated in 1% TTC solution in PBS (pH 7.4) for 5 min at 37°C and then fixed in 10% formalin solution (pH 7.0) for 20–24 hr. TTC stains viable tissue a deep red color, and nonstained tissue is presumed to be infarcted. The images of the slice were captured by a LEICA digital camera 480, and infarct area in each slice was measured by computed planimetry with an image analyzing program ImageJ 1.26 (Wayne Rasband, National Institutes of Health, USA). Then the infarct area of each slice was determined by manual delineation of TTC-negative pale area of the image. The infarct weight of each slice was calculated by multiplying the ratio of infarct area within total area by the slice weight. The infarct weight of each slice was summed to produce the total infarct weight of each left ventricle. Finally, the infarct size was calculated by dividing the total infarct weight of each left ventricle by the total weight of the ventricle [16].

### 2.7. Measurement of Nitrate/Nitrite, LDH, and CK

Total nitrate/nitrite concentrations of serum from six time points in I/R were measured in a simple two-step process by using a colorimetric assay. In brief, sera were ultrafiltered through a 30 KDa molecular weight cut-off filter using Amicon Ultra-4 Centrifugal Filter Units (Millipore, USA) that reduces background absorbance due to the presence of hemoglobin and improves color formation using the Griess reagents. The filtrates were then subjected to a nitrate/nitrite colorimetric assay (Cayman Chemical Company, USA) with a spectrophotometer (Bio-RAD, USA) at 540 nm according to the procedures recommended by the manufacturer. Serum activities of lactate dehydrogenase (LDH) and creatine kinase (CK-MB) were determined by using commercial assay kits according to manufacturer's recommendations.

### 2.8. Western Blot Analysis

Western blot analyses of total proteins and phosphorylated form of proteins in hearts were performed according to methods described previously with modifications. In brief, frozen heart tissue was homogenized using a homogenizer (IKA, Germany) in ice-cold lysis buffer (Sucrose 250 mM, Tris-HCl (pH 7.2) 50 mM, sodium EDTA 2 mM, beta-mercaptoethanol 2 mM, sodium fluoride 5 mM, sodium orthovanadate 1 mM, aprotinin 10 *μ*g/mL, leupeptin 10 *μ*g/mL) for 5 min. The homogenate was immediately centrifuged at 14,000 g for 20 min at 4°C; the supernatant was gently collected and stored at −80°C until use. The contents of total protein in the supernatants were determined using a Bio-Rad kit (Bio-Rad Laboratories, Hercules, CA, USA). Equal amounts of protein were boiled in sample loading buffer for 5 min, loaded on 10% SDS polyacrylamide gel, and finally transferred onto Immobilon-P membrane (Pore size: 0.45 *μ*m, Millipore, USA). The nonspecific binding sites on the membrane were blocked with 5% nonfat milk in Tris-buffered saline plus 0.1% Tween-20 (TBST) at 4°C overnight. Then the membranes were incubated with specific primary antibodies at 18°C for 3 hours; the antibodies were anti-PI3 kinase p85, anti-phospho-PI3 kinase p85 (Tyr458), anti-Akt1/PKBa, anti-phospho-Akt1/PKBa (Ser473), anti-eNOS/NOSIII, anti-phospho-eNOS (Ser1177), and anti-GAPDH (Cell Signaling Technology or BD Transduction Laboratories, USA). Membranes were subsequently incubated with peroxidase-conjugated secondary antibodies (Bio-Rad Laboratories, USA) in 5% nonfat milk in TBST for 1 hr at room temperature. The membranes were then washed six times, and the immunoreactive proteins were detected by enhanced chemiluminescence (ECL) method using hyperfilm and ECL reagent (Amersham, USA) according to the manufacturer's instructions. Band intensities were quantified using a densitometer analysis system (Quantity One software, Bio-Rad).

### 2.9. Measurement of Testosterone in Serum

Serum testosterone level was assayed using commercially available ELISA kits according to the manufacturer's instructions (CALBIOTECH, INC., Austin Dr, Spring Valley, CA). One milliliter venous blood sample from each rat was drawn at four time points of 0, 60, 90, and 180 minutes. These blood samples were centrifuged at 1,000 g and 4°C for 20 minutes; serum stored was stored at −80°C for 2 weeks before assay.

### 2.10. Statistical Analysis

Data were expressed as mean ± S.E.M. One-way ANOVA with multiple comparisons using the Student-Newman-Keuls test was used to analyze differences between groups. All statistical analysis was performed using SPSS 16.0 software (SPSS, Inc., Chicago, IL, USA). Differences were considered significant if *P* < 0.05.

## 3. Results

### 3.1. Myocardial Infarct Size after Myocardial I/R 

**Figure 3:**
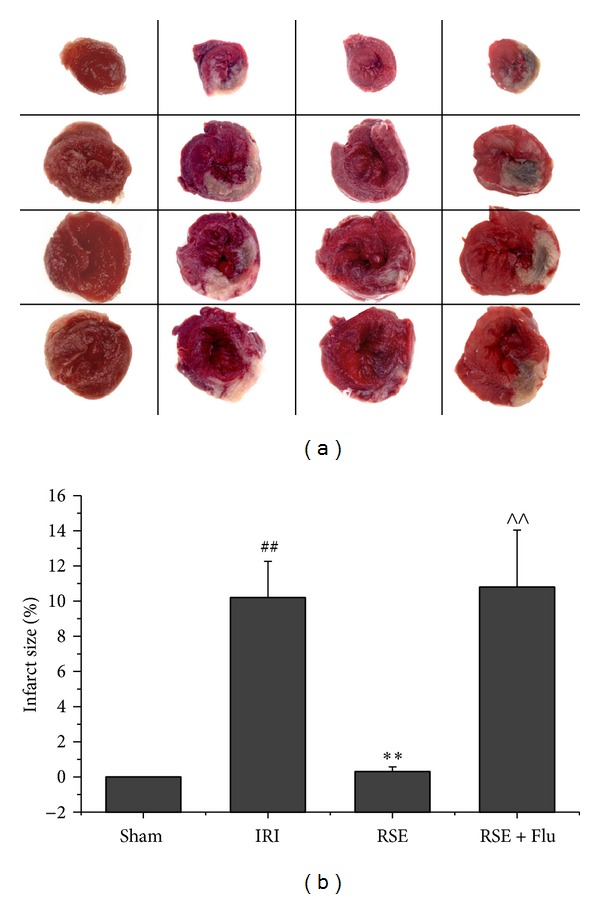
Infarct size after myocardial I/R injury. (a) Representative photos of TTC stained left ventricle slices. Deep red-staining areas indicate normal tissue, and unstained pale areas indicate infarct tissue. (b) Bar chart of myocardial infarct size determined by TTC staining. Data are shown as mean ± S.E.M, *n* = 6~7/group. ^##^ *P* < 0.01 versus Sham group, **P < 0.01 versus IRI group, ^∧∧^ *P* < 0.01 versus RSE group.

Occluding and releasing the LAD coronary artery of SD rats is a widely used model for antimyocardial I/R injury research. The severity of I/R injury can be assessed by measuring the size of infarct area (myocardial infarct size). As shown in Figure 3, the myocardial infarct size produced by 30 min ischemia and 90 min reperfusion in IRI group was 10.5 ± 2.20%. Orally given 60 min before the acute ischemia, RSE at 80 mg/kg exhibited significant protection to rat hearts against I/R injury with an infarct size of 0.3 ± 0.32% (compared with IRI group, *P* < 0.01). Combined administration of RSE and flutamide (at 80 mg/kg and 10 mg/kg, resp.) did not induce any significant reduction in myocardial infarct size (10.7 ± 3.56%, *P* < 0.05 and *P* < 0.01, compared with IRI or RSE group, resp.). This clearly indicates that flutamide abolished the protective effect of RSE against I/R injury.

### 3.2. Serum CK-MB and LDH Activities after Myocardial I/R

**Figure 4:**
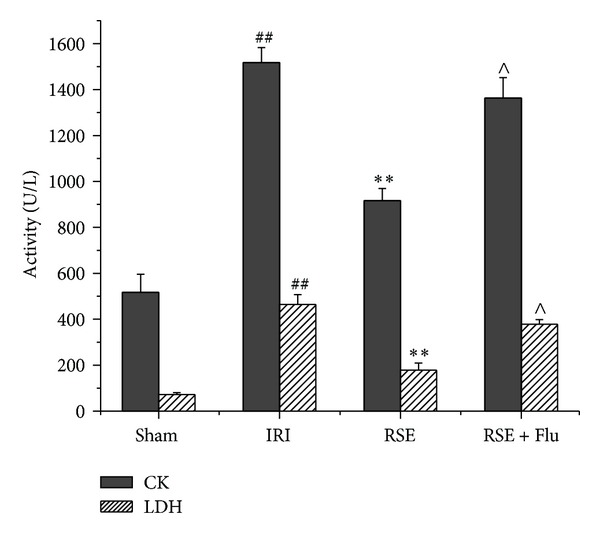
Effects of RSE and flutamide on CK and LDH activities in serum after myocardial I/R. The unit of enzymes was expressed as U/L. Data are shown as mean ± S.E.M, *n* = 5/group. ^##^ *P* < 0.01, versus Sham group; **P < 0.01 versus IRI group; ^∧^ *P* < 0.05 versus RSE group.

Besides the evaluation of infarct size induced by I/R, we also measured myocardial enzymes in serum in order to further elucidate the effect of RSE with or without flutamide in rat open chest model. As shown in Figure 4, the activities of CK-MB and LDH in the serum of rats that underwent myocardial I/R (IRI group) were both significantly increased when compared with the sham group without I/R (CK-MB activity: 1476 ± 137 U/L versus 522 ± 214 U/L, *P* < 0.01; LDH activity: 435 ± 85 U/L versus 67 ± 16 U/L, *P* < 0.01). Compared with the IRI group, 80 mg/kg RSE treatment significantly inhibited the elevation of CK-MB (894 ± 134 U/L, *P* < 0.01) or LDH (178 ± 69 U/L, *P* < 0.01) activities induced by acute I/R injury. The joint use of RSE at 80 mg/kg and flutamide at 10 mg/kg did not significantly suppress the increase of serum CK-MB or LDH activity (1363 ± 199 U/L, 378 ± 45 U/L, compared with IRI group *P* > 0.05, *P* > 0.05, resp.). These results also revealed that flutamide significantly abolished the beneficial effect of RSE on the myocardial enzymes (compared with RSE group, CK-MB, *P* < 0.05 and LDH, *P* < 0.05) in I/R injury. 

### 3.3. Time Course Changes of Serum NO Production (Nitrate and Nitrite) during Pretreatment and I/R Period

**Figure 5:**
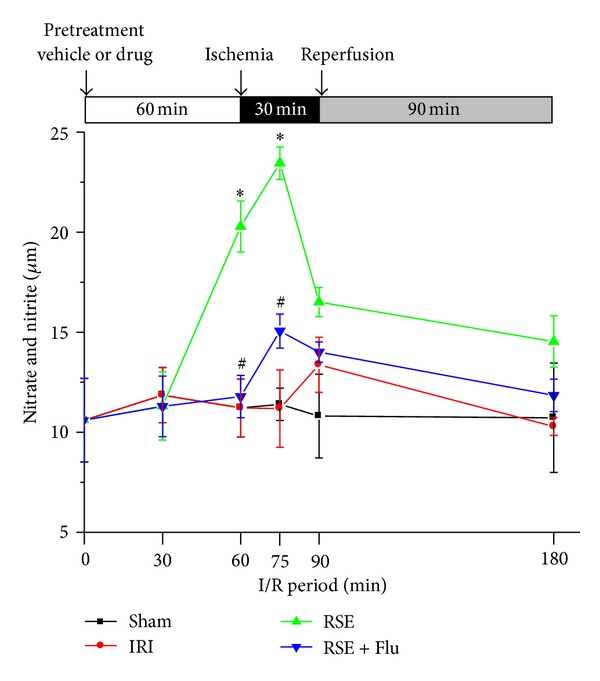
Effects of RSE and flutamide on time course changes of serum NO production. The concentrations of NO in serum were determined by measuring serum nitrite and nitrate levels. Data are shown as mean ± S.E.M, *n* = 3~5/group at each time point. *P < 0.05, RSE group versus IRI group at the corresponding time point; ^#^ *P* < 0.05, RSE + Flu group versus RSE group at the corresponding time point.

During acute myocardial I/R injury, nitric oxide (NO) plays important physiological and pathological roles in a time-dependent mechanism. We found that oral administration of RSE significantly protected rat hearts against I/R, and this effect was abolished by joint administration of flutamide. We wondered whether this protective effect of RSE involves an NO-related mechanism that is somehow affected by flutamide. We first measured the NO level in the serum of rats that underwent sham surgery or I/R process without drug pretreatment. The result showed no significant difference in the serum level of NO between the sham group and the IRI group. However, pretreatment of RSE in rats that underwent I/R process resulted in a remarkable elevation of serum NO production. NO production increased after 30 min RSE administration and reached a peak at 15 min after ischemia (compared with IRI group, *P* < 0.05).  Figure 5 illustrates these time-dependent changes in serum NO production during pretreatment and I/R period. In the RSE + Flutamide group, the levels of NO production were significantly lower than those in RSE group (*P* < 0.05 at 1 h after pretreatment and *P* < 0.05 at 15 min after ischemia), suggesting that flutamide did indeed suppress NO production stimulated by RSE treatment.

### 3.4. Time Course Changes of PI3K/AKT/eNOS Signaling Pathway in Heart Tissue during Pretreatment and I/R Period

**Figure 6:**
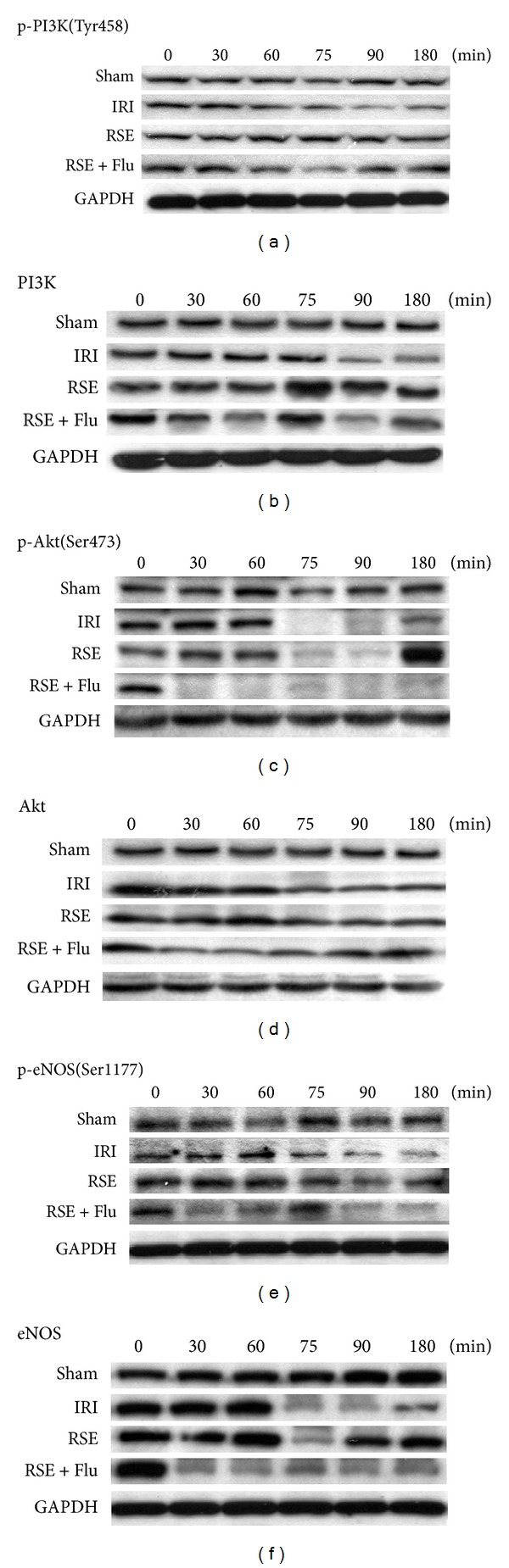
Effects of RSE and flutamide on time course changes of activation and expression of PI3K/Akt/eNOS signaling. PI3K/Akt/eNOS signaling in heart was investigated by Western blot at six time points. The blot was reprobed sequentially with antibodies special for PI3K or phospho-PI3K (Tyr458), Akt or phospho-Akt (Ser473), or eNOS or phospho-eNOS (Ser1177). Identical results were obtained in three separate rat hearts at each time point. Normalization of Western blot was ensured by GAPDH.

Since the NO production during I/R injury is closely regulated by the PI3K/AKT/eNOS signaling pathway, we further investigated the influence of RSE with or without joint administration of flutamide on this pathway during pretreatment and I/R period.  Figure 6 illustrates representative results of the effects of RSE and flutamide on expression and activation of PI3K/Akt/eNOS pathway at the different time points during pretreatment and I/R period. Total and active PI3K, Akt, and eNOS were approximately equivalent in the sham group throughout the pretreatment and I/R periods. I/R significantly suppressed the expression and activation of PI3K after 30 min ischemia (Figures 6(a) and 6(b)). Expression of Akt in the IRI group was reduced slightly after ischemia, while its activation (p-Akt) was dramatically inhibited after ischemia (Figures 6(c) and 6(d)). Total eNOS in IRI group was decreased after ischemia and gradually returned to normal levels after reperfusion. However, the p-eNOS was constantly decreased compared with the sham group (Figures 6(e) and 6(f)). RSE pretreatment returned PI3K back to a normal level and further upregulated activation of PI3K during the 30 min ischemia period. Although RSE did not significantly change the total form of Akt, it significantly elevated the p-Akt at 90 min after reperfusion. Meanwhile, in the presence of RSE pretreatment, active and total eNOS decreased remarkably at 15 min after ischemia and returned to normal level at 90 min after reperfusion. In the RSE and flutamide joint administration group, p-Akt dramatically and continuously decreased at 30 min after pretreatment. Interestingly, the expression and activation of eNOS in myocardial tissue was significantly reduced at 30 min after pretreatment. Combined with flutamide, RSE pretreatment did not show any obvious influence on returning total or active form of eNOS to normal levels during the I/R period. These results strongly suggest that the anti-ischemia reperfusion injury effect of RSE might result from, or at least involves, PI3K/Akt-activated eNOS activation in the early phase of ischemia (15 min after ischemia). This result is also partly in accordance with the results of NO production assay. 

### 3.5. Time Course Change of Serum Testosterone Level during Pretreatment and I/R Period

**Figure 7:**
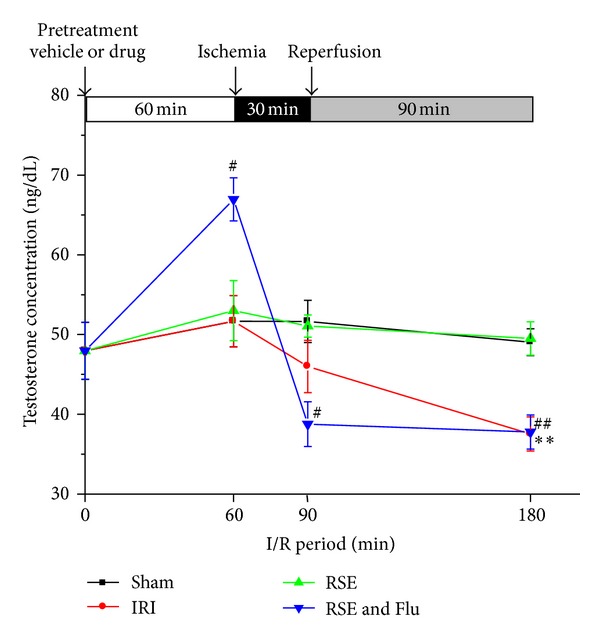
Effects of RSE and flutamide on time course changes of serum testosterone concentration. Data are shown as mean ± S.E.M, *n* = 3~5/group at each time point. ^#^ *P* < 0.05, ^##^ *P* < 0.01, RSE + Flu group compared with Sham group at the corresponding time point; **P < 0.01 IRI group compared with Sham group at the corresponding time point.

As shown in Figure 7, the level of testosterone was stable in the sham group during the whole pretreatment and I/R periods. Although 30 min ischemia did not cause a significant decrease in serum testosterone, additional 90 min reperfusion resulted in a significant decrease (23.5% drop compared with the sham group, *P* < 0.01). Pretreatment with RSE completely suppressed the effect of I/R on serum testosterone levels. Interestingly, after 60 minutes pretreatment, the RSE + Flutamide group showed a significant increase (*P* < 0.05) in serum testosterone, but this effect was dramatically reversed after 30 minutes of ischemia (*P* < 0.05) and this reversion remained to the end of 90 min reperfusion. 

## 4. Discussion 

In the rat open chest model, the hearts of rats that received RSE alone or both RSE and flutamide exhibited great difference in myocardial injury after acute ischemia reperfusion. We demonstrated for the first time an increased susceptibility to myocardial I/R injury after blocking androgen receptor in treatment of ginseng. Administering flutamide interrupted myocardial tissue recovery, as demonstrated by a significantly larger myocardial infarct size and higher myocardial enzyme activities. These effects could partly be explained by lower NO production, which leads to decreased cardiomyocytes' protective effect triggered in the early phase of ischemia as well as in the following reperfusion period. Furthermore, relationship between changes of PI3K/Akt/eNOS signal and endogenous testosterone level was time dependently associated with the contrasting effects on recovery from ischemia in ginseng with or without flutamide. These findings illustrate that, during I/R, androgen receptor blockage by flutamide abolished the effectiveness of ginseng on acute myocardial I/R injury. This action was closely related to inhibition of PI3K/Akt/eNOS signaling and increase of endogenous androgen levels. 

Sex differences exist in the response of myocardium to acute ischemia injury [17]. Clinical trials have found evidence of coronary heart disease in premenopausal women at much lower rate than in age-matched men [18]. Studies on experimental animals have demonstrated multiple beneficial effects of estrogens on the cardiovascular system, including amelioration of ischemia- and reperfusion-induced myocardial injury [15]. Therefore most previous studies have focused on the cardioprotective role of female hormones, whereas the role of male hormones in this significant gender difference in cardiovascular health has been largely neglected. Recently the role of androgen plays in the development of cardiovascular disease has attracted increasing interest. Testosterone, the most important endogenous androgen, has been considered as a vasoactive sex hormone steroid. Current results showed that testosterone may relax vascular smooth muscle by endothelium-dependent or -independent mechanisms, which may in part explain why testosterone replacement therapy improves myocardial ischemia in patients with coronary artery disease [16]. Thus, supplemental testosterone has been used to treat men with angina. The beneficial effects of supplemental testosterone on both ischemia and exercise tolerance have been demonstrated partially in several in vivo and in vitro studies. Administration of androgen was able to cause coronary dilatation and improve exercise-induced myocardial ischemia in male patients in men with established coronary artery disease [19]. This kind of androgen hormone supplement may be related to direct coronary vasodilation by NO-dependent and -independent pathways, and its activity does not seem to be dependent on conversion into estrogens. Most importantly, these possible mechanisms explain why the androgen hormone has been shown to improve angina pectoris in patients who received hormone replacement clinically. Therefore, these effects have led to the view that androgens are beneficial for the IHD. 

On the contrary, more and more evidence shows that anabolic androgenic steroids are associated with myocardial ischemia, sudden cardiac death, and hypertension in athletes, suggesting that androgens may negatively affect myocardial tolerance of ischemia [20]. Androgenic steroids consist of a variety of different steroids with differing pharmacological properties. Among them, the incidence of cardiovascular morbidity closely associated with testosterone has been widely investigated [21]. Endogenous testosterone in men declines with age, and administering anabolic testosterone supplements to elderly men seems to improve their cardiovascular health [22]. And the use of testosterone esters like testosterone enanthate in males induces transient super-physiological androgen levels [23]. One study has shown that a single acute exposure to exogenous testosterone before ischemia worsened myocardial function recovery and increased activation death signaling [24]. Another study also supported that endogenous testosterone has a negative effect on myocardial function and Akt signaling [25]. These findings imply that testosterone can have negative effects on the myocardium after acute injury. Thus, evaluation of the different roles of endogenous and supplemental testosterone for cardiovascular health is of growing importance.

Furthermore, many studies have shown that testosterone has an important role in cardiac injury because the heart can accumulate testosterone at higher concentrations than other androgen target organs, and functional androgen receptors are present in isolated cardiac myocytes [26]. Testosterone also has been demonstrated to modulate nuclear transcription by membrane receptor second messenger cascades, including L-type calcium channel, NO-dependent or NO-independent mechanisms [27], and apoptotic cell death of cardiomyocytes [28]. Another evidence also shows that the non-genomic pathway of androgen action possibly involves targeting of membrane hormone receptors to functional signaling modules in membrane caveolae, enabling the rapid activation of PI3K/Akt kinase pathways and endothelial nitric oxide synthase [29]. However, in these studies, the testosterone-regulated signaling of PI3K/Akt kinase remained unclear. 

Recent reports on the cardioprotective effects of ginseng indicate that the upregulation of eNOS and NO production activity in I/R injury hearts by ginseng are possibly important mechanisms [30]. Our previous studies demonstrated that standardized ginseng extract protected rat hearts against ischemia and reperfusion injury in a dose-dependent manner. Oral treatment with RSE 1 h before ischemia significantly reduced myocardial I/R injury. This quick effect was possibly produced by increasing NO production. And this protection against I/R injury can be partially explained by active compounds of ginseng with sex hormone-like activities. Ginsenosides Rb1 and Rg1 have been shown to induce NO production and increase eNOS activation in aortic endothelial cells [31]. Activation of the steroid hormone receptors, such as ER and GR, through nongenomic and/or genomic pathways represents an important mechanism underlying the cardiovascular protective effect of ginseng therapy [32]. Therefore, we proposed to investigate whether there is possibly an association between the cardioprotective effect of ginseng and the androgen-mediated PI3K/Akt kinase signaling pathway. We applied flutamide, which is a known androgen-receptor blocker, and investigated whether blocking the androgen receptor might influence the protective effect of ginseng on the myocardial ischemia reperfusion injury in an *in vivo* rat heart model. In addition, because NO-dependent mechanism is of major importance for ischemia-reperfusion injury, we also used time-dependent relationship to assess the PI3K/Akt/eNOS signaling pathway during myocardial I/R.

The main findings of this study are that the androgen receptor blocker (flutamide) (1) abolished the antimyocardial I/R injury effect of *Panax ginseng*, (2) decreased NO production and enhanced CK-MB and LDH activities, (3) inhibited the expression and/or activation of PI3K/Akt/eNOS signaling pathway, and (4) exhibited a direct effect on endogenous testosterone level during myocardial I/R. Our present study provides new evidence that endogenous androgen-mediated action and downstream functional signaling modules are involved in the cardioprotective effect of *Panax ginseng*. 

## Abbreviations

IHD: Ischemic heart disease
I/R: Ischemia reperfusion
NO: Nitric oxide
TTC: 3,5-Triphenyltetrazolium chloride
RSE: Standardized ginseng extract
LDH: Lactate dehydrogenase
CK-MB: Creatine kinase.
